# Potential of a Novel Patient-Reported Outcome Assessment Method Utilizing Illustrations in Rheumatoid Arthritis

**DOI:** 10.7759/cureus.79473

**Published:** 2025-02-22

**Authors:** Kensuke Koyama, Ryousuke Koizumi

**Affiliations:** 1 Department of Rheumatology, Nirasaki City Hospital, Nirasaki, JPN; 2 Department of Orthopedic Surgery, University of Yamanashi, Chuo, JPN

**Keywords:** clinical disease activity index, health assessment questionnaire, illustration, okomarigoto sheet, patient reported outcome, rheumatoid arthritis, simplified disease activity index

## Abstract

Objectives: We investigated whether the "Okomarigoto sheet," a novel patient-reported outcome measure that utilizes illustrations, correlates with disease activity and the Health Assessment Questionnaire Disability Index (HAQ-DI) in rheumatoid arthritis.

Methods: The Okomarigoto sheet comprises five items each for morning stiffness, pain, and fatigue. Individual items are scored as follows: severe symptoms = 2, mild symptoms = 1, no symptoms = 0. The total score ranges from 0 to 30; higher scores indicate greater symptom burden. Correlations between Okomarigoto sheet scores, disease activity scores, and the HAQ-DI were analyzed in 332 patients. Scores were compared between patients in remission (HAQ-DI ≤ 0.5) and not in remission.

Results: Correlations between the total Okomarigoto sheet score and Disease Activity Score using 28-joint count C-reactive protein, Clinical Disease Activity Index, and Simplified Disease Activity Index were 0.488, 0.591, and 0.572, respectively. The correlation between the total Okomarigoto sheet score and HAQ-DI was 0.593. The mean Okomarigoto sheet score was significantly lower in patients achieving HAQ-DI remission compared to those not in remission (2.47 vs. 10.4, respectively; p < 0.01).

Conclusions: This novel patient-reported outcome measure performed comparably to existing measures, correlating strongly with disease activity and disability.

## Introduction

The Japan College of Rheumatology (JCR) guidelines emphasize that the primary goals of the treatment of rheumatoid arthritis (RA) are to improve long-term prognosis by enhancing the quality of life (QOL) and extending life expectancy through reducing disease activity and inhibiting joint destruction [1]. Key international guidelines concur, stating that the ultimate therapeutic objective for patients with RA is to achieve and maintain clinical remission [1-3]. With the establishment of these treatment goals and advancements in evaluation methodologies, treatment outcomes have improved progressively [4]. Recently, a growing interest has focused on patient-reported outcome measures (PROMs), which assess the enhancement of QOL, a crucial secondary treatment goal.

In RA, the most widely used PROMs include the Health Assessment Questionnaire-Disability Index (HAQ-DI) [5], Short Form Health Survey-36 (SF-36) [6], EuroQol-5D (EQ-5D) [7], and Modified Health Assessment Questionnaire (MHAQ) [8]. These tools are typically administered as self-reported questionnaires. While certain scales, such as the Visual Analog Scale (VAS) and the Pediatric Quality of Life Inventory (PedsQL), incorporate facial scales to assess symptom severity [9], none of the available PROMs utilize visual illustrations as a core component of symptom assessment.

The "Okomarigoto sheet" (OS) is a novel PROM that incorporates illustrations and was developed in collaboration with both rheumatologists and patients with RA [10]. It consists of five items each for morning stiffness, pain, and fatigue, with symptoms categorized as severe, mild, or absent. The unique use of illustrations and onomatopoeia in the OS is intended to help patients recognize and articulate their symptoms more clearly. The use of illustrations is a particularly innovative feature compared to existing PROMs. However, a significant limitation of the OS is its current availability exclusively in Japanese, which constrains the generalizability of research findings. Furthermore, the relationship between OS scores, RA disease activity, and existing PROMs has not yet been evaluated. This study aimed to investigate the association between OS, measures of RA disease activity, and HAQ-DI. Additionally, as a secondary objective, we examined whether OS scores varied based on the achievement of HAQ-DI remission.

This study was presented as a conference abstract at the 35th Chubu Rheumatology Meeting held on September 6, 2024.

## Materials and methods

Okomarigoto sheet (OS)

**Figure 1:**
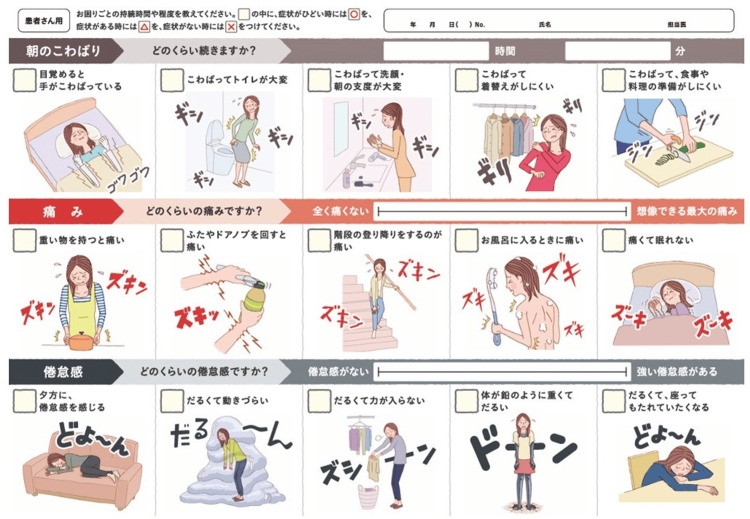
Okomarigoto sheet (OS). The OS can be accessed via the Japanese College of Rheumatology (JCR) website (http://www.ryumachi-jp.com/general/checksheet/). This sheet is presented  with permission from the JCR.

The OS can be accessed via the Japanese College of Rheumatology (JCR) website (http://www.ryumachi-jp.com/general/checksheet/). This sheet is presented in Figure 1 with permission from the JCR. Each of the five items that evaluate morning stiffness, pain, and fatigue includes an illustration accompanied by a brief explanation, though the OS is currently only available in Japanese. In this study, the OS score was defined as follows: morning stiffness, pain, and fatigue were assessed across five questions, with scoring criteria as follows: severe symptoms = 2, mild symptoms = 1, and absence of symptoms = 0. The total OS score ranged from 0 to 30, with higher scores indicating a greater burden of RA symptoms.

Patients

This study was a single-center, cross-sectional study of patients with RA who were treated at our hospital alone between January and April 2024. We evaluated 332 patients with RA, all of whom met the 2010 RA classification criteria [11], and exclusion criteria included patients with dementia who were unable to complete the questionnaire, as well as those presenting with symptoms of infectious disease on the day of evaluation.

This study was approved by the Ethics Committee of Nirasaki City Hospital (Approval No. 7-1). Written informed consent was obtained from all patients in accordance with the Declaration of Helsinki.

Data collection

Demographic and clinical data were collected from electronic medical records and included age, sex, disease duration, anti-cyclic citrullinated peptide antibody (ACPA) positivity, rheumatoid factor (RF) positivity, use of methotrexate (MTX), biologic/targeted synthetic disease-modifying antirheumatic drugs (b/ts DMARDs), glucocorticoid (GC) usage, swollen joint count (SJC) and tender joint count (TJC) of 28 joints, patient global assessment (PGA), physician global assessment (Ph-GA), serum C-reactive protein (CRP), HAQ-DI [5], Disease Activity Score using 28-joint count CRP (DAS28-CRP) [12], Clinical Disease Activity Index (CDAI) [13], Simplified Disease Activity Index (SDAI) [14], and OS score.

HAQ-DI

The HAQ-DI comprises 20 questions categorized into eight domains: dressing, rising, eating, walking, hygiene, reaching, gripping, and performing errands or chores [5]. Each question is rated on a four-level difficulty scale ranging from 0 to 3, where 0 indicates no difficulty, 1 denotes some difficulty, 2 represents considerable difficulty, and 3 signifies inability to perform the task. The HAQ-DI score is calculated as the mean of the highest scores within each of the eight categories, yielding a possible range of 0 to 3.

DAS28-CRP

We computed a modified version of the DAS28-CRP [12] using the following formula:

DAS28-CRP = (0.56 × √TJC) + (0.28 × √SJC) + (0.36 × ln [CRP (mg/L) + 1]) + (0.014 × PGA [mm]) + 0.96,

where DAS28-CRP = Disease Activity Score using 28-joint count-C-reactive protein, TJC = tender joint count of 28 joints, SJC = swollen joint count of 28 joints, and PGA = patient global assessment.

CDAI

The CDAI integrates multiple parameters of RA disease activity by computing a linear sum of the tender and swollen joint counts (0-28 scale), and patient and evaluator global assessments of disease activity (each rated on a 0-10 cm visual analog scale) [13].

SDAI

The SDAI integrates multiple parameters of RA disease activity by computing a linear sum of the tender and swollen joint counts (0-28 scale), patient and evaluator global assessments of disease activity (each rated on a 0-10 cm visual analog scale), and the CRP level (measured in mg/dL) [14].

Statistical analysis

First, we assessed the correlations between OS scores and DAS28-CRP, CDAI, and SDAI. Second, we examined the correlation between OS scores and HAQ-DI. Last, we compared OS scores between patients in HAQ-DI remission (REM; defined as HAQ-DI ≤ 0.5) and those not in remission (NREM). Correlations between OS scores, disease activity, and HAQ-DI were evaluated using Spearman's rank correlation coefficient. The Mann-Whitney U test was used to assess statistical differences. All statistical analyses were conducted using GraphPad Prism, version 9.0 (GraphPad Software, San Diego, CA, USA). Statistical significance was set at p < 0.05.

## Results

Patient characteristics

The characteristics of the 332 patients included in this study are summarized in Table 1. The median age was 69.5 years, with a median disease duration of 10 years. Most (74.7%) were female individuals, 74.4% were RF positive, and 73.5% were ACPA positive. MTX was used by 63.9% of patients, while b/ts DMARDs were used by 44.3%. The specific b/ts DMARDs used included tocilizumab (72 patients), etanercept (30 patients), abatacept (15 patients), golimumab (six patients), baricitinib (six patients), ozoralizumab (five patients), infliximab (four patients), upadacitinib (four patients), adalimumab (three patients), sarilumab (one patient), and peficitinib (one patient). The median DAS28-CRP, CDAI, and SDAI scores were 1.6, 2.5, and 2.79, respectively, and the median HAQ-DI score was 0.06.

**Table 1:** Patient characteristics. RF, rheumatoid factor; ACPA, anti-citrullinated protein antibody; MTX, methotrexate; b/ts DMARDs, biologic/targeted synthetic disease modifying antirheumatic drugs; CRP, C-reactive protein; DAS28-CRP, disease activity score using 28-joint count-CRP; CDAI, Clinical Disease Activity Index; SDAI, Simplified Disease Activity Index; HAQ-DI, Health Assessment Questionnaire-Disability Index; OS, Okomarigoto sheet, IQR, interquartile range.

Characteristics	n=332
Age, y, Median (IQR 25%,75%)	69.5 (58, 76)
Sex, Female (%)	74.7
Disease duration, y, Median (IQR 25%,75%)	10 (5, 16)
RF positive, %	74.4
ACPA positive, %	73.5
Steinbrocker Stage, I/II/III/IV (%)	35.5/42.8/19/2.7
Steinbrocker Class, I/II/III/IV (%)	47.6/41.9/9.6/0.9
MTX use, %	63.9
MTX dose, mg/week, Median (IQR 25%,75%)	8 (6, 10)
Glucocorticoid use, %	19.0
Glucocorticoid dose, mg/day	1 (1, 1.5)
b/ts DMARDs use, %	44.3
Swelling Joint Counts, Median (IQR 25%,75%)	0 (0, 0)
Tender Joint Counts, Median (IQR 25%,75%)	0 (0, 1)
Patient Global Assessment, Median (IQR 25%,75%)	1 (0.5, 3)
Physician Global Assessment, Median (IQR 25%,75%)	1 (0.5, 2)
CRP, mg/dL, Median (IQR 25%,75%)	0.06 (0.02, 0.19)
DAS28-CRP, Median (IQR 25%,75%)	1.6 (1.2, 2.22)
CDAI, Median (IQR 25%,75%)	2.5 (1, 6)
SDAI, Median (IQR 25%,75%)	2.79 (1.08, 6.10)
HAQ-DI, Median (IQR 25%,75%)	0.06 (0, 0.375)
Total OS Score, Median (IQR 25%,75%)	2 (0, 5)

Correlations between OS score and RA disease activity scores

The correlations between the total OS score and DAS28-CRP, CDAI, and SDAI were 0.488, 0.591, and 0.572, respectively, indicating strong associations. The correlations between the morning stiffness (MS) score and DAS28-CRP, CDAI, and SDAI were 0.419, 0.495, and 0.486, respectively, also demonstrating strong correlations. The correlations between the pain score and DAS28-CRP, CDAI, and SDAI were 0.508, 0.582, and 0.569, respectively, showing high correlations. However, the correlations between the fatigue score and DAS28-CRP, CDAI, and SDAI were weaker, at 0.296, 0.386, and 0.371, respectively (Table 2).

**Table 2:** Correlation between RA disease activity scores and total OS score and subdomain scores. RA, rheumatoid arthritis; OS, Okomarigoto sheet; MS, morning stiffness; DAS28-CRP, disease activity score using 28-joint count- C-reactive protein; CDAI, Clinical Disease Activity Index; SDAI, Simplified Disease Activity Index.

	DAS28-CRP		CDAI		SDAI	
	r	p	r	p	r	p
Total OS score (r, 95% CI)	0.488 (0.40 to 0.57)	<0.001	0.591 (0.51 to 0.66)	<0.001	0.572 (0.49 to 0.64)	<0.001
MS score (r, 95% CI)	0.419 (0.32 to 0.51)	<0.001	0.495 (0.41 to 0.57)	<0.001	0.486 (0.40 to 0.57)	<0.001
Pain score (r, 95% CI)	0.508 (0.42 to 0.59)	<0.001	0.582 (0.50 to 0.65)	<0.001	0.569 (0.49 to 0.64)	<0.001
Fatigue score (r, 95% CI)	0.296 (0.19 to 0.39)	<0.001	0.386 (0.29 to 0.48)	<0.001	0.371 (0.27 to 0.46)	<0.001

Correlation between OS score and HAQ-DI score

The correlation between the total OS score and HAQ-DI was 0.593, indicating a strong association. The correlations between HAQ-DI and the MS score, pain score, and fatigue score were 0.508, 0.616, and 0.384, respectively (Table 3).

**Table 3:** Correlation between HAQ-DI and total OS score and subdomain scores. OS, Okomarigoto sheet; MS, morning stiffness; HAQ-DI, Health Assessment Questionnaire-Disability Index.

	HAQ-DI	
	r	p
Total OS score (r, 95% CI)	0.593 (0.52 to 0.66)	<0.001
MS score (r, 95% CI)	0.508 (0.42 to 0.59)	<0.001
Pain score (r, 95% CI)	0.616 (0.54 to 0.68)	<0.001
Fatigue score (r, 95% CI)	0.384 (0.29 to 0.48)	<0.001

Comparison of OS scores between patients in HAQ-DI remission and not in remission

**Figure 2:**
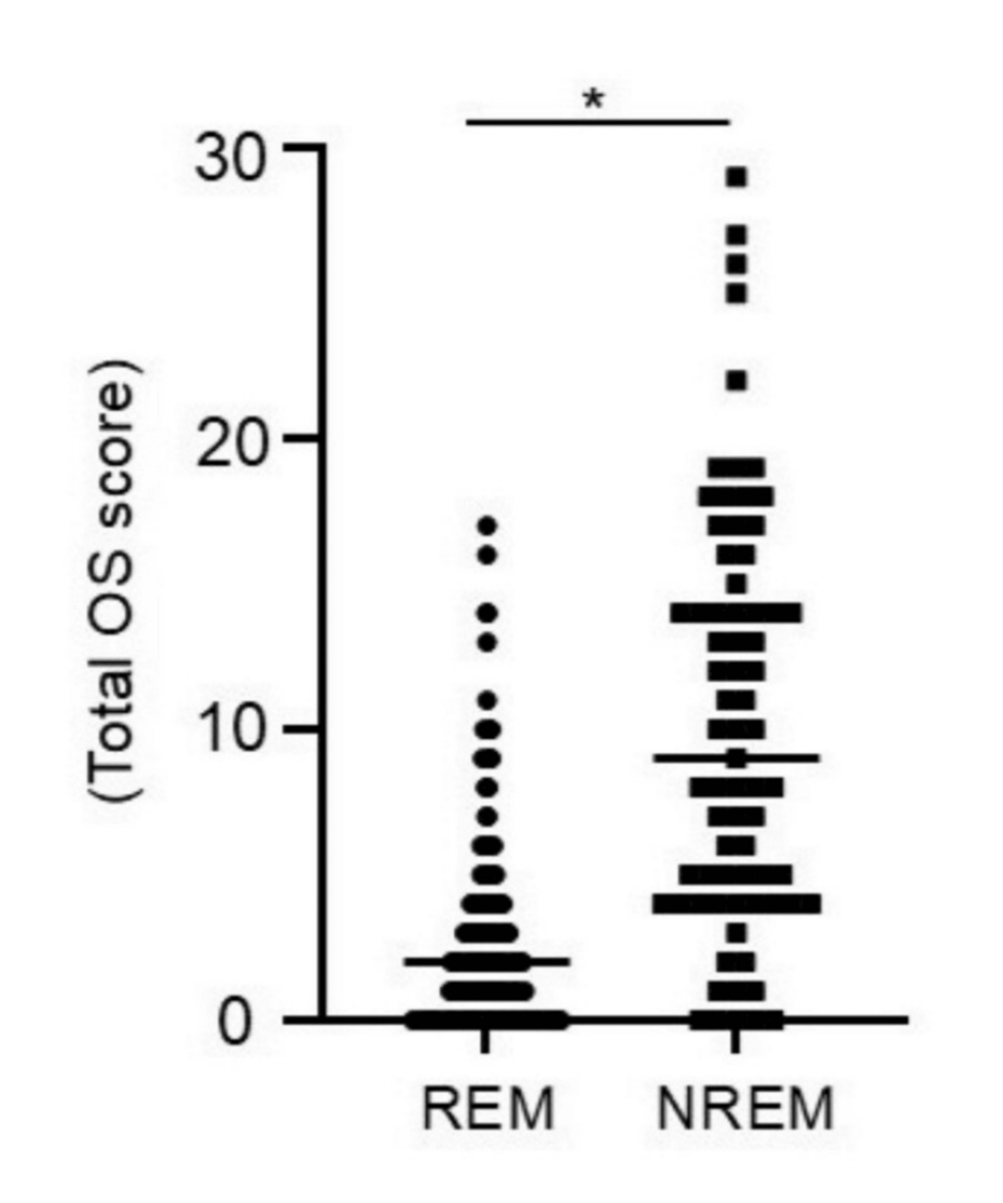
Comparison of OS scores between patients in HAQ-DI remission and not in remission. The mean total OS score was 2.47 in the HAQ-DI remission group and 10.4 in the non-remission group, with significantly lower OS scores observed in the HAQ-DI remission group (Mann-Whitney U test, p < 0.01). REM, remission; NREM, not in remission; OS, Okomarigoto sheet; HAQ-DI, Health Assessment Questionnaire-Disability Index.

HAQ-DI remission (REM) was achieved in 259 patients, while 73 patients were not in remission (NREM). the mean total OS score was 2.47 in the REM group and 10.4 in the NREM group, with significantly lower OS scores observed in the REM group (Figure 2).

## Discussion

In this study, we evaluated whether the OS, a novel patient-reported outcome (PRO) assessment tool that utilizes illustrations, correlates with RA disease activity and HAQ-DI. Our findings indicated a strong correlation between the OS and established composite disease activity measures as well as the HAQ-DI, suggesting the potential of OS as a new PRO assessment tool for RA. To the best of our knowledge, this is the first report demonstrating the feasibility of OS as a PRO tool for RA.

Previous studies have reported a generally strong correlation between RA disease activity scores and PROs, with correlations between the HAQ-DI and the DAS28, CDAI, and SDAI ranging from 0.45 to 0.47 [15]. Moreover, the time-averaged HAQ-DI has shown even stronger correlations with the time-averaged DAS28, CDAI, and SDAI, with values of 0.64, 0.69, and 0.70, respectively [16]. For instance, the Routine Assessment of Patient Index Data 3 (RAPID3) assessment correlates with the DAS28, with values ranging from 0.555 to 0.701, and with the CDAI, with values ranging from 0.761 to 0.843 [17,18]. In contrast, some reports suggest that while the SF-36 correlates with the DAS28, it does not correlate well with the CDAI or SDAI [19]. In the present study, the total OS score showed correlations with RA disease activity scores, ranging from 0.488 to 0.591, which are comparable to other established PROMs.

With regard to functional impairment, strong correlations have been documented between the HAQ-DI and SF-36 physical function (r = 0.75-0.79) and social function (r = 0.49-0.61) [20,21], pain visual analog scale (VAS) scores (r = 0.71), Rheumatoid Arthritis Quality of Life Scale (RAQoL) (r = 0.81), EQ-5D (r = -0.79) [22], and MHAQ (r = 0.856-0.88) [21]. In our study, the total OS score correlated with the HAQ-DI at r = 0.59, which suggests that the OS is as useful as other PROMs. However, some PROMs, such as the SF-36 mental scale score (r = 0.35-0.36) and the Arthritis Impact Measure (AIMS) psychological score (r = 0.33), have shown only weak correlations with HAQ [20]. Similarly, the OS fatigue score showed a weaker correlation with HAQ-DI (r = 0.384), reflecting a comparable trend. Fatigue assessment using the Functional Assessment of Chronic Illness Therapy-Fatigue (FACIT-F) questionnaire [23] has been reported to strongly correlate with poorer HAQ-DI scores [24]. As the validity of the OS score has not yet been established, its correlation requires further evaluation in future studies.

When comparing the groups in HAQ-DI remission and not in remission, previous studies have reported better RA disease control in the HAQ-DI remission group [16,25]. Although the HAQ-DI is commonly used as a final outcome measure, few studies have examined the differences in other PROMs between patients in HAQ-DI remission and those not in remission. In this study, OS scores were generally lower in the HAQ-DI remission group; however, a subset of patients in that group exhibited high OS scores. We believe that further investigation of this subset could provide valuable insights for improving the management of patients with RA.

Limitations

This study has several limitations. First, the OS score was defined based on severe symptoms scoring 2, mild symptoms scoring 1, and no symptoms scoring 0, with total scores ranging from 0 to 30. However, the scoring system has not yet been validated, and the results could vary if the scoring definitions were adjusted. Second, 116 cases (50%) in this study had a HAQ-DI score of 0, suggesting a potential floor effect. Nonetheless, as a HAQ-DI of 0 represents an optimal outcome for patients with RA, we included all such cases in our analysis. Among individuals with an OS score of 0, 79.8% had a HAQ-DI score of 0. The relationship between OS scores and HAQ-DI scores should be reexamined in a cohort with a more uniform distribution of HAQ-DI scores. However, even when the HAQ-DI score is 0, a nonzero OS score suggests the presence of unmet needs [26,27] and residual symptoms [28], indicating that OS might capture aspects insufficiently assessed by existing PROMs. Third, the OS is currently available only in Japanese, and the use of illustrations combined with onomatopoeia raises concerns about whether it would be interpreted consistently outside of Japan. However, the OS utilizes illustrations and onomatopoeia to facilitate the visualization of symptoms, offering the advantage of enabling respondents to provide intuitive answers. In addition, another clinical benefit is that the survey requires only a few minutes to complete.

## Conclusions

This novel RA PROM utilizing illustrations demonstrated strong correlations with RA disease activity and the HAQ-DI, comparable to existing PROMs. While challenges remain, including the need for multicenter validation and adaptation for use in languages other than Japanese, the OS shows promise as a potential new method for evaluating PROMs in RA. Further studies are warranted to establish its broader applicability and reliability across diverse settings and languages.
